# Optimization of Machining Parameters to Minimize Cutting Forces and Surface Roughness in Micro-Milling of Mg13Sn Alloy

**DOI:** 10.3390/mi14081590

**Published:** 2023-08-12

**Authors:** Ali Ercetin, Kubilay Aslantaş, Özgür Özgün, Mustafa Perçin, Manjunath Patel Gowdru Chandrashekarappa

**Affiliations:** 1 Department of Naval Architecture and Marine Engineering, Maritime Faculty, Bandırma Onyedi Eylul University, Bandırma 10200, Turkey; 2 Department of Mechanical Engineering, Faculty of Technology, Afyon Kocatepe University, Afyonkarahisar 03200, Turkey; aslantas@aku.edu.tr; 3 Department of Occupational Health and Safety, Faculty of Health Sciences, Bingöl University, Bingöl 12000, Turkey; oozgun@bingol.edu.tr; 4 Department of Machine and Metal Technologies, Vocational School of Technical Sciences, Bursa Uludag University, Bursa 16059, Turkey; mustafapercin@uludag.edu.tr; 5 Department of Mechanical Engineering, PES Institute of Technology and Management, Visvesvaraya Technological University, Belagavi 590018, India; manjunath.mech@pestrust.edu.in

**Keywords:** micro-milling, cutting forces, feed rate, surface roughness, ploughing, material removal rate (MRR)

## Abstract

This comprehensive study investigates the micro-milling of a Mg13Sn alloy, a material of considerable interest in various high-precision applications, such as biomedical implants. The main objective of the study was to explore the optimizations of variable feed per tooth (fz), cutting speed (Vc), and depth of cut (ap) parameters on the key outcomes of the micro-milling process. A unique experimental setup was employed, employing a spindle capable of achieving up to 60,000 revolutions per minute. Additionally, the study leveraged linear slides backed by micro-step motors to facilitate precise axis movements, thereby maintaining a resolution accuracy of 0.1 μm. Cutting forces were accurately captured by a mini dynamometer and subsequently evaluated based on the peak to valley values for Fx (tangential force) and Fy (feed force). The study results revealed a clear and complex interplay between the varied cutting parameters and their subsequent impacts on the cutting forces and surface roughness. An increase in feed rate and depth of cut significantly increased the cutting forces. However, the cutting forces were found to decrease noticeably with the elevation of cutting speed. Intriguingly, the tangential force (Fx) was consistently higher than the feed force (Fy). Simultaneously, the study determined that the surface roughness, denoted by Sa values, increased in direct proportion to the feed rate. It was also found that the Sa surface roughness values decreased with the increase in cutting speed. This study recommends a parameter combination of fz = 5 µm/tooth feed rate, Vc = 62.8 m/min cutting speed, and ap = 400 µm depth of cut to maintain a Sa surface roughness value of less than 1 µm while ensuring an optimal material removal rate and machining time. The results derived from this study offer vital insights into the micro-milling of Mg13Sn alloys and contribute to the current body of knowledge on the topic.

## 1. Introduction

Magnesium (Mg) alloys are a class of lightweight materials that have been increasingly used in a variety of applications, particularly in the automotive and aerospace industries because of their high strength-to-weight ratio [1]. These alloys are composed primarily of magnesium, the lightest structural metal, combined with other elements such as aluminum, zinc, silicon, copper, or rare earth elements to improve its properties [2,3]. Mg alloys are approximately 33% lighter than aluminum, 60% lighter than titanium, and 75% lighter than steel [4]. This lightness, combined with adequate strength, makes them attractive for industries where weight reduction is a priority [5].

In the world of biomedical science, magnesium (Mg) alloys have come into the spotlight as an attractive material option. This growing interest is predominantly due to their unique properties of biocompatibility and bioresorbability [6]. The ability of these alloys to interact safely with biological systems without causing adverse reactions makes them an excellent choice for use within the human body [7,8]. Furthermore, their bioresorbable nature, which allows them to degrade and be absorbed by the body over time, provides a significant advantage for certain medical applications [6,9].

Specifically, the slow degradation of Mg alloys in the physiological environment has catalyzed extensive research in their potential use for temporary implantable devices [10,11,12]. Examples of such devices are screws employed for the fixation of bone fractures [13,14]. The premise here is to harness the bioresorbability of Mg alloys to create implants that can naturally dissolve in the body over a specified period. This process eliminates the requirement for these implants to be surgically removed, therefore reducing the need for additional surgeries. The potential to minimize the risks, costs, and discomfort associated with secondary surgeries represents a significant advancement in patient care [15,16,17].

Despite these promising attributes, there are challenges to consider when it comes to the manufacturing of these biomedical Mg alloys. Given that many of these implants, such as fracture fixation screws, are relatively small in size, their fabrication calls for precision and intricacy [18,19,20]. This necessity places micromachining into an essential role in the fabrication process of these biomedical Mg alloys [21,22].

The Mg13Sn alloy has garnered substantial attention in the biomedical field, particularly in the context of implants [23]. The alloy’s unique mechanical properties, combined with its high biocompatibility and excellent biodegradability, make it an ideal candidate for orthopedic and other types of implants [24]. A significant area of research regarding this alloy is its machining properties, including milling [25,26,27]. Machining processes, such as milling, play a critical role in the manufacture of implants, shaping the alloy into precise geometries necessary for various applications [28].

Micro-machining technologies based on tools, including micro-milling, micro-turning, micro-grinding, and micro-drilling, operate on principles similar to those of traditional machining processes [29,30,31,32]. Mechanical micro-machining processes provide flexibility, making it possible to fabricate miniature components with intricate geometries, regardless of the material’s nature. The process thus promises versatility and precision in crafting micro-components for biomedical applications [31].

Despite the potential applications and benefits, the micro-milling of biomedical Mg-Sn alloys remains significantly understudied, leaving a gap in knowledge that is crucial for refining the fabrication process [33,34]. This deficit could be hindering the full exploration of Mg-Sn alloys’ capabilities and their extensive use in the biomedical field [35,36,37].

To address this gap, our study delves into an investigation of optimum cutting parameters for the micro-milling of Mg13Sn alloys. We apply different cutting speeds, feed rates, and depths of cut parameters to understand how these factors can impact the efficacy and roughness parameters of micro-milled surfaces. In this article, we will provide insights into our investigation and discuss potential implications and improvements for the micromachining of biomedical Mg-Sn alloys.

## 2. Materials and Methods

In this study, Mg13Sn alloy produced by the spark plasma sintering method was used as the workpiece material. The detailed fabrication process for the production and characterization of the alloy by spark plasma sintering is available in [38]. The compositions and properties of Mg13Sn alloy is given in Table 1.

**Figure 1:**
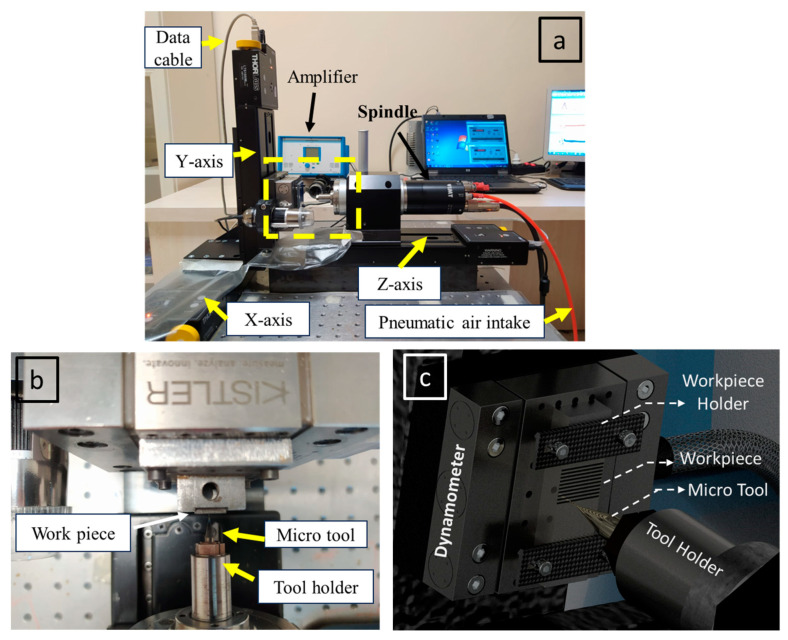
(**a**) Micro-milling experimental setup; (**b**) close-up view of the cutting test zone; and (**c**) schematic presentation of cutting test zone.

Figure 1 introduces a micro-milling experimental arrangement, close-up view of the cutting test zone, and a schematic depiction of the cutting test zone, conceived for executing micro-machining at elevated cutting speeds. Capable of achieving up to 60,000 revolutions per minute, the spindle’s revolution settings can be finely tuned with the aid of computer software specially designed for this purpose. The spindle, which also sets the cut’s depth, moves along the z axis. The fixture housing the workpiece is mounted over a mini dynamometer (Kistler-9119AA1), responsible for capturing cutting force data and conveying it to the computer system. Axis movements have been facilitated by linear slides backed by micro step motors. The feed direction of the workpiece has been designated as the x axis. Each axis in the experimental configuration maintains a resolution accuracy of 0.1 μm.

**Figure 2:**
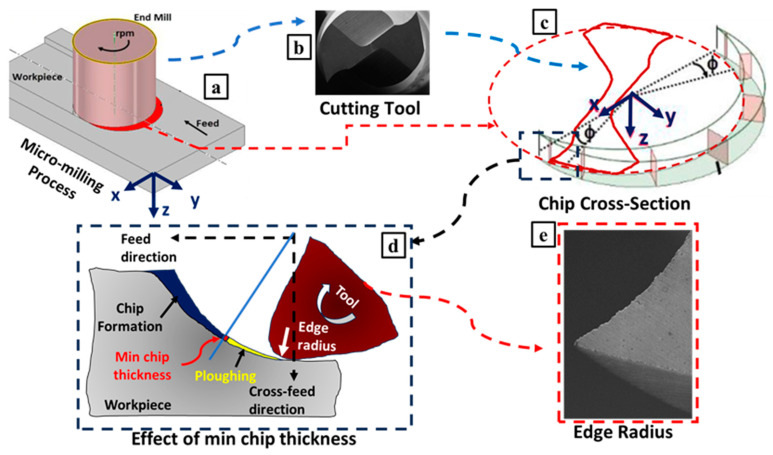
(**a**) Micro-milling process; (**b**) cutting tool; (**c**) the variation in chip cross-section; (**d**) effect of min chip thickness; and (**e**) edge radius.

Figure 2 showcases the variation in chip cross-section during the micro milling process and includes a schematic diagram illustrating the impact of minimum chip thickness. This experimental setup, akin to a horizontal machining center in its design, leverages pneumatic air pressure to affix the micro cutting tool to the spindle. For the study, two-flute end mills with diameters of 508 µm were employed. In the experiments, uncoated cutting tools obtained from Performance Micro Tool company were used. The edge radius of the cutting tool in the micro milling procedure was identified through SEM analysis, employing the LEO 1430 VP model. Measurement outcomes revealed the edge radius to be roughly 1.45 µm (edge radius in Figure 2). General properties of the cutting tool are given in Table 2. In the micro-milling experiments, the feed per tooth (fz), cutting speed (Vc), and depth of cut (ap) were taken as variable to determine the effects of the cutting parameters. The cutting tests have been carried out with different parameters of feed rate, cutting speed, and depth of cut (Table 3). For each application of the feed rate parameter, a constant value was assigned to the cutting speed and depth of cut parameters, as detailed in Table 3. Once a given test was concluded, the subsequent parameter of cutting speed was applied. After the implementation of all the cutting speed parameters, the same procedures were reiterated for the depth of cut, as ap = 400 µm.

**Figure 3:**
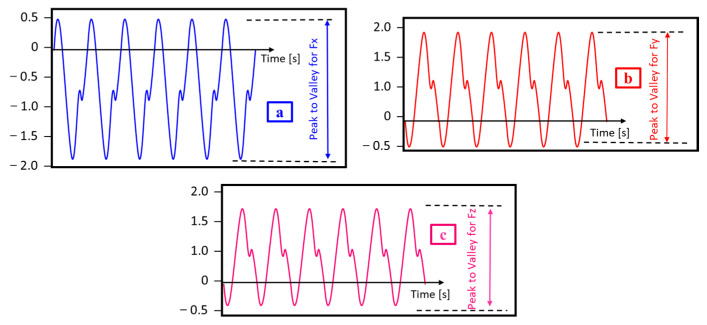
Measurement of cutting forces (**a**) peak to valley for Fx; (**b**) peak to valley for Fy; and (**c**) peak to valley for Fz.

To mitigate the impact of wear on the cutting tool, each test utilized a fresh tool. The cutting force signals were amplified and transduced into force measurements, subsequently recorded to a computer using Dynoware software. Given that the sampling rate for the dynamic cutting force under measurement was 7 kHz, the cutting force signals underwent bandpass filtering within the 300 Hz–7 kHz range. The cutting forces were evaluated based on the peak to valley values for Fx, Fy, and Fz (Figure 3), a common practice in micro-milling cutting tests. These values refer to the amplitudes between the maximum and minimum values. As for the cutting force measurements, the cutting distance was set at 30 mm. A Nanovea-ST400 3D Optical profilometer was employed to measure surface roughness. By performing surface scan along the width of the slot, the areal surface roughness (Sa) of the slot was acquired.

## 3. Results and Discussion

### 3.1. Cutting Forces

**Figure 4:**
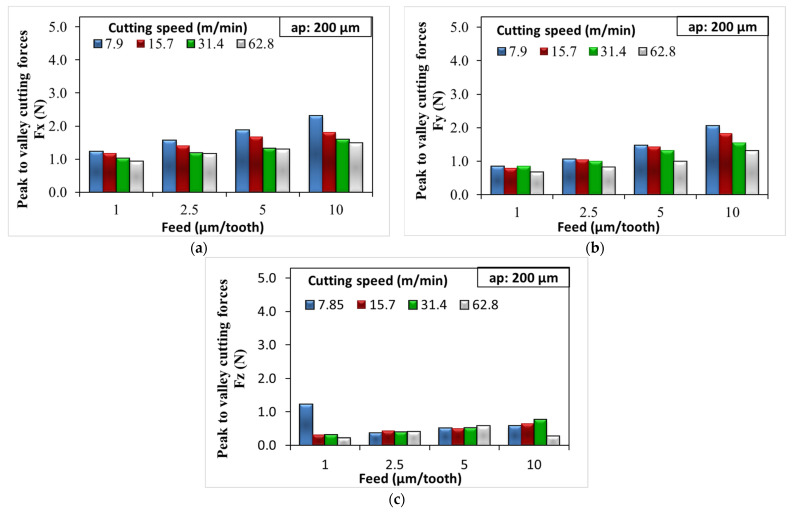
Cutting forces obtained at different feed rates and 200 µm depth of cut: (**a**) Fx; (**b**) Fy; and (**c**) Fz.

**Figure 5:**
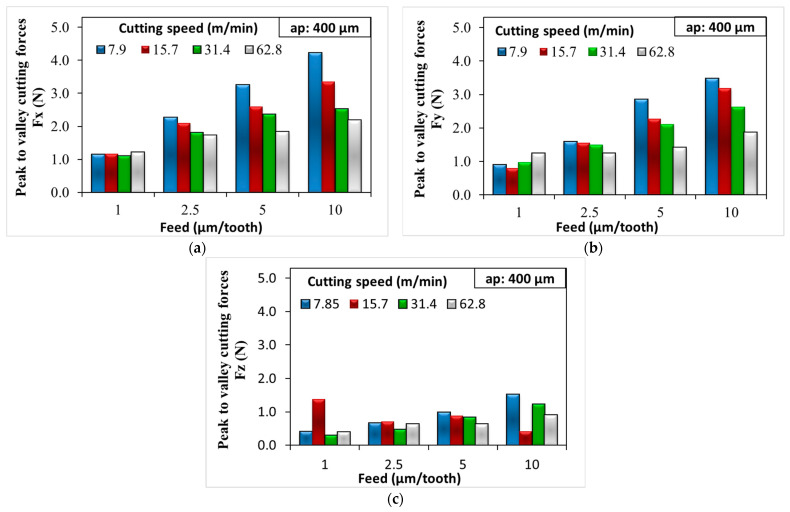
Cutting forces obtained at different feed rates and 400 µm depth of cut: (**a**) Fx; (**b**) Fy; and (**c**) Fz.

The Fx, Fy, and Fz forces resulting from the micro-milling parameters applied at 200 µm and 400 µm depth of cut are given in Figure 4 and Figure 5, respectively. When Figure 4 and Figure 5 are analyzed together, it is seen that increasing feed rate and depth of cut significantly increase the cutting forces Fx and Fy. When the feed and depth of cut are increased, the tool engages with more material during each revolution or pass. This requires more force, which, in turn, increases the cutting forces. In essence, increasing the feed rate and depth of cut makes the tool cut more material in the same amount of time, thereby increasing the load on the tool.

However, in contrast to this situation, there is a significant decrease in cutting forces in micro-milling experiments where the cutting speed increases. Cutting speed denotes the velocity at which the cutting edge of the tool engages the workpiece. Elevated cutting speeds can lead to a rise in temperature within the cutting zone, which, in turn, induces thermal softening of the workpiece [39,40]. The heat produced in this zone can play a role in further diminishing the cutting forces. This effect is especially pronounced during micro-milling operations, given that the cross-section of the chip is exceedingly small. In a study of Saglam et al. [41], it was observed that cutting speed and cutting forces share an inverse relationship; an increase in the cutting speed results in a reduction in the cutting force. However, this increased speed also resulted in a higher temperature at the tool tip.

**Figure 6:**
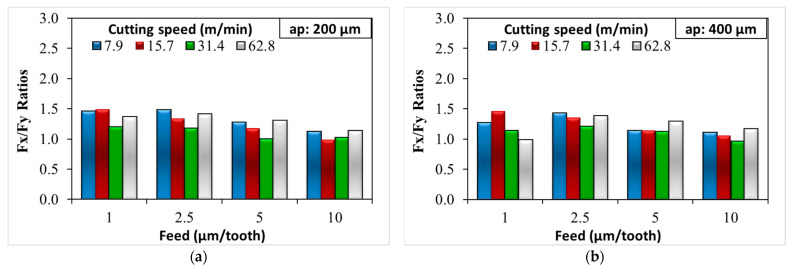
Fx/Fy ratios for (**a**) 200 µm depth of cut and (**b**) 400 µm depth of cut.

An additional outcome emerged from our studies; the Fx cutting force values obtained in the application of the micro milling process at both 200 µm and 400 µm depth of cut were higher than the Fy cutting forces. Fx is typically larger because it is aligned with the primary direction of material removal. It has to overcome the shear strength of the material being cut, which requires significant force. On the other hand, the Fy is typically smaller, as it is primarily overcoming friction between the tool and the material rather than directly causing material deformation or removal. In Figure 6, the Fx/Fy ratio decreases with both increasing feed rate and increasing cutting speed. For the tests with 200 µm depth of cut parameter, the max and min Fx/Fy ratios were 1.48 and 0.98, respectively (Figure 6a). For the tests where a 400 µm depth of cut parameter was applied, the max and min Fx/Fy ratios were 1.45 and 0.95, respectively (Figure 6b). When the feed rate increased, the thickness of the chip also increased, causing an increase in both Fx and Fy. However, the Fy force generally increased more rapidly than the Fx cutting force. This was because a larger feed rate meant more material was engaged per tool revolution, which result in a larger feed force. The minimum cutting force value was obtained at a depth of cut of 200 µm, a feed rate of 1 µm/tooth, and a cutting speed of 62.8 m/min. The maximum cutting force value was obtained at 400 µm, 10 µm/tooth feed rate, and 7.9 m/min cutting speed.

Our examination has led to another significant result. When the cutting forces for the fz = 1 µm/tooth feed parameter in Figure 5a are analyzed, it is possible to say that the increase in cutting speed has no significant effect on the cutting forces. At low feed rates, the tool did not remove much material with each revolution (the chip load is low). This means that even though the tool is interacting with the workpiece more times per unit time (because of the increased cutting speed), the amount of material being removed in each interaction is minimal. As a result, the cutting forces do not increase significantly. The mechanics underlying chip formation in the micro-machining process differ from those in conventional machining. This distinction is due to various factors such as the size effect, tool run-out, and elastic recovery of the workpiece material. Therefore, the phenomenon in the present study is more likely to occur when milling soft materials that do not require significant force to cut, like soft plastics or certain soft metals [42,43,44].

By increasing the depth of cut from 200 µm to 400 µm, there was a natural increase in all cutting forces (Figure 4 and Figure 5). Increasing the depth of cut meant that more material was engaged with the tool during each pass. As such, a larger volume of material was being deformed and removed, which generally required more force [45]. In experiments where the cutting speed was low and the feed rate was high, the cutting forces Fx and Fy could increase by about 3–4 times (Figure 5a,b). Therefore, a minimum cutting speed of 31.4 m/min was more appropriate in terms of the cutting forces obtained. In order to minimize the machining time, the maximum depth of cut ap = 400 µm was more suitable. It was better to select the feed rate as high as possible and determine the optimum feed rate fz according to the surface roughness results.

### 3.2. Surface Roughness

**Figure 7:**
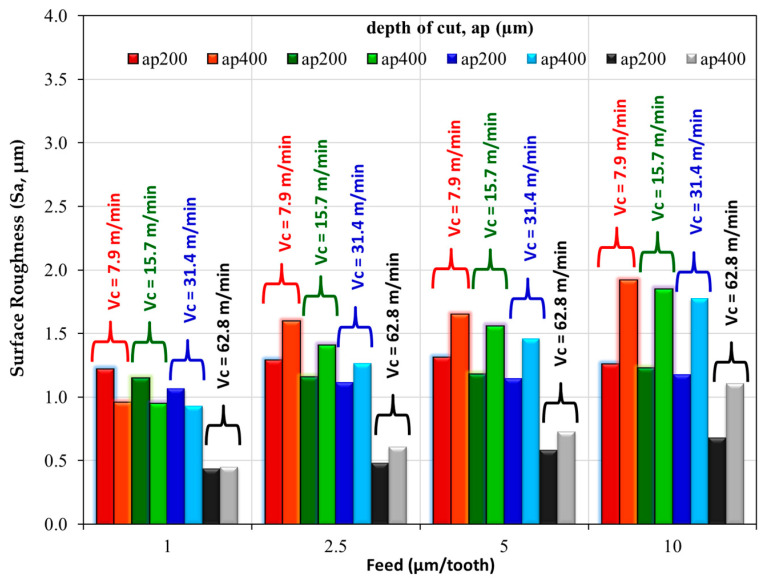
Comparing the surface roughness values of 200 µm and 400 µm depth of cut.

**Figure 8:**
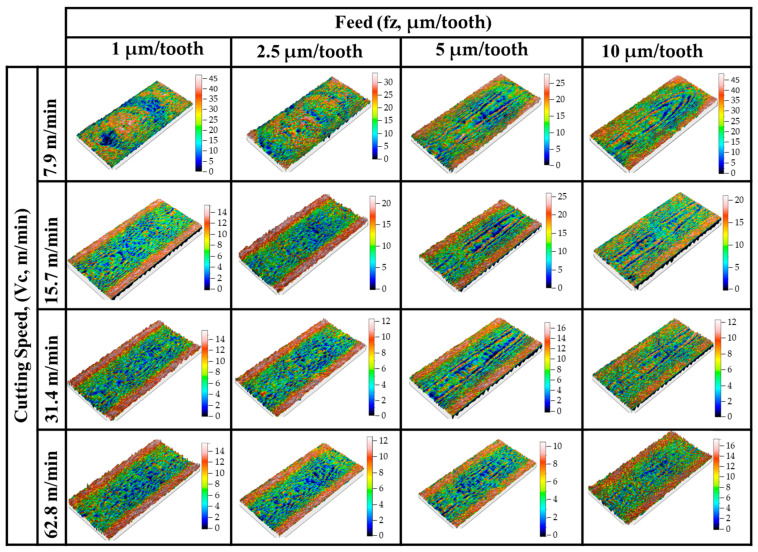
Three-dimensional profilometer images for 200 µm depth of cut.

**Figure 9:**
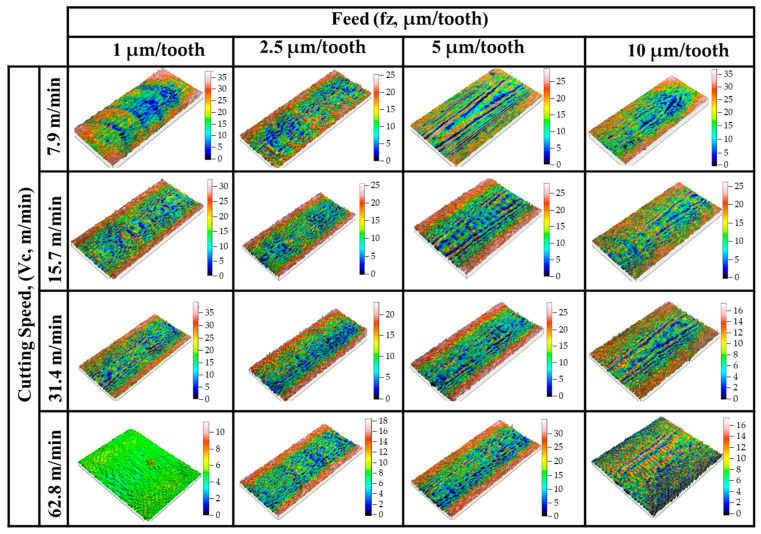
Three-dimensional profilometer images for 400 µm depth of cut.

Comparative surface roughness values of 200 µm and 400 µm depth of cut are given in Figure 7. Three-dimensional profilometer images for 200 µm and 400 µm depth of cuts are given in Figure 8 and Figure 9, respectively. Sa surface roughness values for 200 µm depth of cut increase proportionally with the feed rate. These increases were more noticeable at intermediate parameters with a cutting depth of 200 µm and a cutting speed of 62.8 m/min. However, with all cutting parameters where a cutting depth of 400 µm was applied, Sa surface roughness values significantly increased along with the increasing feed rate. It was thought that the primary reason behind the increase in Sa surface roughness values with an increase in feed was due to the higher material removal rate. Studies have reported that an increase in feed rate directly correlates with an elevation in surface roughness, concluding that this effect is due to a corresponding rise in the material removal rate, which ultimately enhances the surface roughness [46,47,48]. When the feed rate (the rate at which material is fed into the cutting tool) is increased, it leads to a larger volume of material being cut away per unit of time. This increased removal rate causes more fluctuations on the surface profile, resulting in higher surface roughness. In contrast to the effect of feed rate, it is possible to say that Sa surface roughness values decreased significantly with increasing cutting speed (Figure 7). The observed decrease in Sa surface roughness values as cutting speed increases can be largely attributed to the corresponding decrease in the formation of built-up edges (BUEs) [49]. Therefore, a decrease in BUE formation due to higher cutting speeds translates into a smoother surface finish and, consequently, lower Sa surface roughness values. This phenomenon is of utmost importance in precision machining, where achieving low surface roughness is often a key objective.

The surface images clearly exhibit the effects of ploughing when feeds per tooth for both depth of cut parameters were applied at the lowest cutting speed (fz = 1 µm/tooth, Vc = 7.9 m/min parameters in Figure 8 and Figure 9). These irregular cutting marks are a consequence of the tool’s sharp edge endeavoring to sever the chip during the subsequent revolution when it failed to do so in the previous one. As the feed value escalates, there is a noticeable reduction in the irregular marks on the machined surface, and the disparity between the peak and trough narrows. The topography scale provided aptly demonstrates this trend [32].

In the biomedical field where Mg alloys are used for implants such as screws or plates, a smooth surface finish is usually desirable. This is to prevent the adhesion of bacteria and to ensure compatibility with body tissues. In such cases, a surface roughness of less than 1 µm is often targeted. Minimizing the machining time is possible by applying the cutting speed, feed rate, and depth of cut parameters as much as possible. When the column bars with a Sa value below 1 micron, a depth of cut value of ap = 400 µm, and a minimum cutting speed of 31.4 m/min are examined, it is seen that the fz = 5 µm/tooth feed rate is the most appropriate. Therefore, upon analyzing Figure 7, it becomes apparent that a parameter combination of fz = 5 µm/tooth feed rate, Vc = 62.8 m/min cutting speed, and ap = 400 µm depth of cut is highly effective. This specific set of parameters minimizes micro-milling time, maximizes the material removal rate (MRR) within a shorter timeframe, and maintains a Sa surface roughness value of less than 1 µm.

## 4. Conclusions

This comprehensive study, conducted on the micro-milling of Mg13Sn alloys, has revealed significant insights into the effects of varied cutting parameters on the performance and outcomes of this process. The results underscore the complex interplay between cutting parameters such as feed rate, cutting speed, and depth of cut and their subsequent impacts on cutting forces and surface roughness.

The study unequivocally established that an increase in feed rate and depth of cut led to a corresponding escalation in cutting forces (Fx and Fy) and surface roughness values. Interestingly, the application of the cutting speed together with the parameter minimum feed fz = 1 µm did not have a significant effect on the cutting forces. Additionally, the tangential cutting force (Fx) values in micro-cutting experiments at both 200 µm and 400 µm depth of cut exceeded those of feed forces (Fy). This observation underscores the unique dynamics of micro-machining compared to conventional machining.

Surface roughness, denoted by Sa values, increased with the feed rate. For applications like biomedical implants from Mg alloys, it is suggested that a parameter combination of fz = 5 µm/tooth feed rate, Vc = 62.8 m/min cutting speed, and ap = 400 µm depth of cut to maintain a Sa value of less than 1 µm while optimizing machining time and MRR. Additionally, the specified parameters are also suitable in that the cutting forces are not too high.

The observations derived from this study not only contribute to the current body of knowledge on micro-milling of Mg13Sn alloys but also provide insights for improving micro-machining processes. Further research may help optimize the use of Mg alloys in the biomedical sector, potentially unlocking new ways to use these promising materials. Additionally, a mathematical processing of the experimental results in future work would allow obtaining some empirical mathematical models, with the revelation of new aspects regarding the intensity of the influence exerted by the input factors in the micro-milling process on the size of the cutting force and the roughness of the surface generated by micro-milling.

## Figures and Tables

**Table 1:** The compositions and properties of Mg13Sn alloy [38].

Chemical Composition (wt%)	Density (g·cm^−3^)	Hardness (HV)	Tensile Strength (MPa)
87 wt% Mg 13 wt% Sn	1.898	73.3	157.2

**Table 2:** The properties the of cutting tool.

Geometrical Properties			Characteristic Properties		
Shaft diameter (mm)	3.2	Helix length (mm)	2.3	Coating type	Uncoated
Tool diameter (mm)	0.508	Helix angle, θ (°)	30	Hardness (HV 0.05)	1680
Length (mm)	38.3	Rake angle, α (°)	15	Cutting edge radius (µm)	1–1.5
Flute number	2	Relief angle, γ (°)	6	Chemical composition	92 wt% WC + 8 wt% Co

**Table 3:** Cutting parameters applied in the micro-milling experiments.

Experiment No	Feed, fz (µm/Tooth)	Cutting Speed, Vc (m/min)	Depth of Cut, ap (µm)
1; 2; 3; 4	1	7.9; 15.7; 31.4; 62.8	200
5; 6; 7;8	2.5		
9; 10; 11; 12	5		
13; 14; 15; 16	10		
17; 18; 19; 20	1	7.9; 15.7; 31.4; 62.8	400
21; 22; 23; 24	2.5		
25; 26; 27; 28	5		
29; 30; 31; 32	10		

## Data Availability

Not applicable.
